# Female rats release a trapped cagemate following shaping of the door opening response: Opening latency when the restrainer was baited with food, was empty, or contained a cagemate

**DOI:** 10.1371/journal.pone.0223039

**Published:** 2019-10-01

**Authors:** Magnus H. Blystad, Danielle Andersen, Espen B. Johansen

**Affiliations:** Department of Behavioural Sciences, OsloMet—Oslo Metropolitan University, Oslo, Norway; Radboud University Medical Centre, NETHERLANDS

## Abstract

Research on pro-social rat behaviour is growing within the fields of comparative psychology and social neuroscience. However, much work remains on mapping important variables influencing this behaviour, and there is even disagreement on whether this behaviour is empathetically motivated and correctly labelled pro-social, or whether the behaviour is motivated by social contact. The present study used the helping behaviour paradigm where a rat can release a familiar cagemate from a restrainer. Prior to testing with a trapped cagemate, restrainer door opening was trained and baseline opening latencies when the restrainer was empty or baited with food were established. The findings show that the first-time release occurred sooner than in previous research and that rats used a previously demonstrated response to release the trapped cagemate. Further, rats opened the restrainer door more often and with shorter latencies when the restrainer contained a cagemate than when the restrainer was empty, but less often and with longer latencies than when the restrainer contained food. The test of whether illumination levels affect door-opening included in the study showed no effects.

## Introduction

The importance of empathy is made most salient by its absence or dysfunction as evidenced in disorders like autism [1,2], schizophrenia [3,4] and psychopathy [5,6]. Additionally, empathy dysfunction is found in several other psychopathologies [7]. For non-clinical settings, studies on school bullying indicate that a lack of empathy, or low levels of empathy, is a contributing factor [8,9]. Moreover, the relationship between empathy and bullying might be reciprocal [10]. The term empathy originates from a description of feeling at one with aesthetic experience and was proposed to denote the feeling/understanding of the thoughts and behaviour of others [11]. Psychology has a long tradition of experimentally studying empathy (for instance [12,13]), but the emergence of social neuroscience in the nineties [14] offered novel methods and opened for new lines of research. However, there is no single definition of empathy. Researchers in these fields have operationalised empathy in many different ways [15]. In an attempt to distil operationalizations into a single definition, Brown and colleagues arrived at a definition without a behavioural measure which was unfortunate as behavioural measures are paramount for animal studies [15]. Animal research, on the other hand, has identified some possible behavioural measures, specifically pro-social behaviour, which mirrors studies on pro-social behaviour and empathy in humans [16,17]. Animal studies of pro-social behaviour allow for the employment of neuroscientific techniques necessary for understanding the neurobiological bases of empathy and the aetiology of previously mentioned empathy-affecting disorders [18].

Many species engage in pro-social or helping-behaviour, e.g., dolphins [19], ants [20], rats [21], and a wide array of primates [22]. Neural correlates of different empathy responses in rodents indicate common mechanisms in the central nervous system [23]. Brain imaging studies in humans also support the existence of specific neural structures involved in empathy [24]. Taken together, these different findings on animal helping behaviour and neural correlates of empathy support conserved evolutionary mechanisms of empathically motivated helping-behaviour [25]. However, the findings above do not mean that empathy is expressed to the same degree or in the same manner across species. Indeed, species differing in social and cognitive abilities will most likely display different empathic abilities [26] which are in line with theories regarding empathy as having an evolutionary basis [7] (see a comprehensive review of comparative studies of empathy and sympathy in [27]). Animals that are living in social groups, such as rats, would then be expected to show a higher degree of empathic reactions than solitary species.

Early studies indicated that rats selectively react to the distress of cagemates [28] and also show forms of altruism [29] (but see [30] for early discussions and critique). Studies have shown emotional contagion in rats, where one rat responded with freezing behaviour upon observing another rat exhibiting freezing [31,32]. Social communications have also been observed in rats; they express socially relevant information transmitted through ultrasonic vocalizations (USV) [33] (reviewed in [34]). Rats also acted instrumentally to benefit other rats by sharing food, as long as they experienced no cost themselves [35] and the recipient showed food-seeking behaviour [36].

Helping-behaviour was observed in the experiments of Bartal, Decety & Mason [21] where a free rat released a trapped rat from a Plexiglas tube restrainer. They claimed not only that the rats showed helping behaviour, but that it was motivated by a form of empathy [21]. In follow-up studies to Bartal and colleagues seminal paper, researchers have demonstrated the influence of social learning history, anxiolytics and opioids on the helping behaviour of rats [37–39]. However, rigorous experimental control and detailed knowledge about influencing variables are needed to draw firm conclusions regarding the observations (i.e., empathic motivations and pro-social intentions). This point has been accentuated theoretically by researchers [40] and experimental findings [41–43], and it is now debated whether restrainer opening door to release a cagemate is empathically motivated or is motivated by social contact.

The current study used a slightly modified version of the procedure described by Bartal et al. (2011) and was not to designed to separate between possible empathic motivations or social contact. In fact, in accordance with this uncertainty, we will in the following use the neutral and purely descriptive term “door opening” for restrainer door opening resulting in the release of a trapped cagemate. This neutral term avoids labelling behaviour according to assumed controlling or motivating factors (e.g. prosocial door opening, helping).

The present study aims to control for potential confounding variables overlooked in previous studies, and is based on the procedure developed by Bartal et al. with the following additions/modifications:

1. Rats were required to demonstrate opening for a food reward to ensure that door opening was in the rats’ behavioural repertoire before testing with a trapped cagemate.
2. After exhibiting door-opening with a food-baited restrainer, rats were tested for door-opening with an empty restrainer to show that opening and latency was controlled by the content of the restrainer.
3. Only after completion of key points 1 & 2 were the rats tested with a trapped cagemate.

The inclusion of these procedural steps served three related purposes. First, by shaping responding and demonstrating that door-opening is in the rats' behavioural repertoire prior to testing door opening with a trapped cagemate, the learning of door-openings is not left to “accidental” side effect of exploration. Second, testing for door-opening latency when the restrainer is baited (food) or empty provides essential baseline comparisons for interpreting door-opening latency with a trapped cagemate. Interpreting door opening to release the trapped cagemate as pro-social behaviour is problematic if the latency is the same when the restrainer is empty as when a cagemate is trapped inside. Third, the comparison of door-opening latency when the restrainer contains a cagemate with latency when the restrainer contains food reinforcers offers information about the relative strength (reinforcer value) of the two stimuli (cagemate/food) in controlling behaviour.

Additionally, we did a preliminary investigation of the effect of a change in illumination on door openings. Rats are nocturnal animals, and bright light has been described as anxiogenic inducing a fear response [44] and increasing the fear-related startle reflex [45]. Other rat behaviour is also affected by illumination, e.g. maze exploration [46] and social play [47] in addition to physiological measures [48]. Level of illumination also influences rat behaviour in an open-field test [49–51], which is a larger version of the experimental setup in the current study. Effects of illumination have not previously been investigated in the helping behaviour paradigm, and as no measures of stress are included in the study, the test is only preliminary. Hopefully, it can serve as inspiration for studies that investigate this experimental variable more systematically on a more granular level and with the necessary additional measures of stress.

## Materials and methods

### Subjects and housing

Thirty female Sprague-Dawley rats, 100 days old and weighing 150-200g, were purchased from Janvier, France. The animals were randomly divided into 15 couples and housed in transparent cages (412 x 25 x 25). Cohabitation for 14 days began upon arrival at the animal facility in order to establish cagemate relations between the randomly coupled rats. After the cohabitation period in a single home cage, the rats were housed in separate cages, and one rat from each couple was food-deprived during behavioural training. Food deprivation lasted for a total of 10 days, and daily weighing ensured that no rat lost more than 15% of its free-feeding weight. Rats that were food deprived were given smaller rations of standard chow and housed in adjacent cages to maintain social bonds during food deprivation and the separated living phase. This housing situation avoided depriving both animals of food, but enabled the animals to maintain social vocalization, transmission of odours, and observation of behaviour. Additionally, the rats were given 1 hour per day to socialize in a neutral cage except during the weekend. Following food deprivation, the animals were housed together and given food and water ad lib. One couple was removed from the study due to deviant behaviour caused by incorrect deprivation during the shaping procedure. The study was approved by the Norwegian Animal Research Committee (ID# 7966). All procedures for housing and euthanasia were performed at the Department of Biosciences at the University of Oslo (https://www.mn.uio.no/ibv/english/). Daily inspections by the main author, in addition to the in-house animal technicians and veterinarian ensured the animal welfare. All animals were euthanized with carbon dioxide gas.

### Experimental apparatus and technical equipment

**Fig 1:**
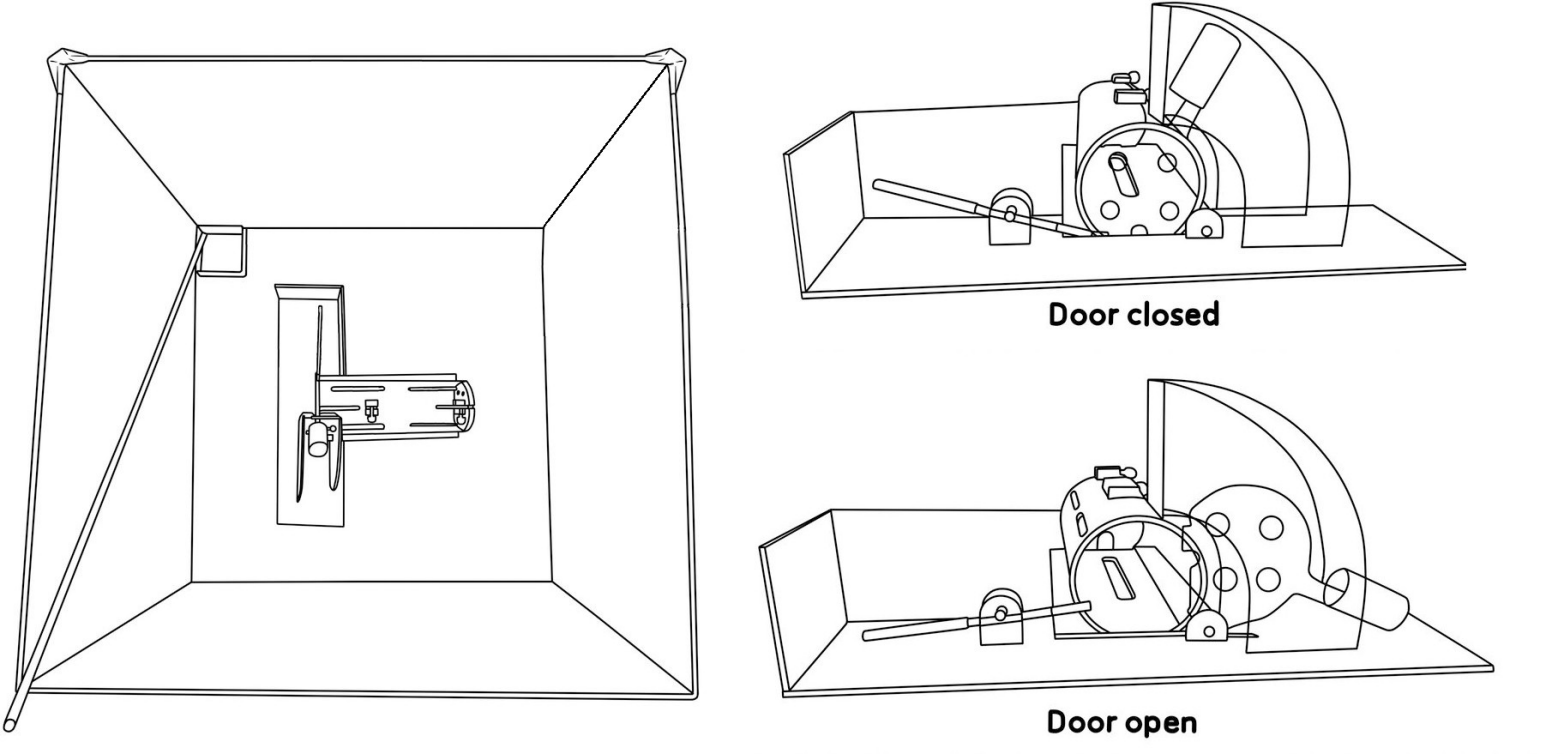
Arena with restrainer. The metal pipe for administration of food reinforcement extended out of the arena on the leftmost side. Right: The door opening mechanism of the restrainer; a lever can be pushed down to tip open a door attached to a counterweight.

The experiment was run in a 0.5*0.5m plastic-glass box, with 0.5m high walls. Matte black duct tape covered all walls to prevent mirror-like reflections. A small metal pipe, extending from the leftmost wall out of the arena and connected to a 5*5cm square metal box positioned in the leftmost corner, was used to administer reinforcers (i.e., food pellets) during magazine training and subsequent behavioural shaping. The experimenters inserted a modified restrainer (Panlab, Harvard Apparatus, Holliston, MS, USA), which is a clear plastic tube with doors on both sides, into the arena during shaping. See Fig 1 for details.

Illumination of the experimental room was measured with a light meter (TES-1337 Digital wd1Light Meter, TES Corporation, Taiwan). Fluorescent ceiling lights were turned on during habituation, the two sessions when the restrainer contained a trapped cagemate (sessions 12 and 13), and during the light phase when testing effects of illumination (sessions 14 and 15). The average and median illuminations were 385 and 407 lux with lights on, and 1 lux with lights off, respectively. These lux values will be referred to as “light” and “dark” through the remainder of the paper. A vertically mounted digital video-camera (Panasonic HC-V160, Panasonic Corporation, Japan) recorded behaviour during testing.

### Procedure

The rats underwent habituation to the arena, magazine training, and three phases of hand shaping using the method of successive approximations:

1. The first phase consisted of location shaping; food was administered when the rats ventured into the quadrant of the arena with the opening mechanism, door and food box.
2. The second phase consisted of more precisely based location shaping; food was administered when the rats spent time by the door opening mechanism.
3. The third phase consisted of presenting food rewards for engagement with the lever, pressing or pushing it down, causing the door to open.

The rats transitioned gradually at individual paces through the three phases. All animals acquired the lever pressing response before proceeding to the next condition testing with food in the restrainer. Both restrainer doors were open for exploration in the first day of shaping. In condition Food through DL, one door was always closed while the other could be opened from the outside using either a lever-press, tipping the door open with paws/head, or tipping over a counterweight. If a trapped rat escaped, a plastic cap was fitted inside the restrainer to prevent access to the door, similar to Bartal et al. (2011). Then, the rat was re-inserted before restarting the trial.

No animals were tested on Saturdays or Sundays. Lights in the animal facilities were on between 7 am to 7 pm. During each session of testing, four trials were run. Training and test-conditions up to session 15 were performed in the light phase of the rats’ ultradian rhythm, as their day-and-night cycle was not inverted in the animal facilities where the animals were kept when not tested. The 15 experimental sessions took place over 15 days, see Table 1 below for an overview of the experimental conditions.

**Table 1:** Overview of experimental conditions across sessions.

Session	Condition	Restrainer content	Description
1	Magazine Training	No restrainer	Habituation to the arena. Response-independent food delivery. Food deprivation, average bodyweight decline was 1.4% (range 0–2%)
7-Feb	Three shaping phases	Empty	Shaping of location in the arena and opening of restrainer door. Food deprivation: average bodyweight decline was 7.8% (range 6–12.7%)
8–10	Food^a^	Food pellets	Food placed inside the restrainer. Food deprivation: average bodyweight decline was 10.6% (range 7.5–14.3%)
11	Empty	Empty	Restrainer empty. No food deprivation, average bodyweight fully recovered^c^ (range 0–1.5%)
12–13	Trapped cagemate Day 1 & 2^b^ (CM1,CM2)	Cagemate	Test for opening restrainer door. No food deprivation, average bodyweight fully recovered^c^ (range 0–2.2%)
14	Light to dark (L-D)	Cagemate	First trial light, then dark-dark-light. No food deprivation, average bodyweight fully recovered^c^ (range 0–0.7%)
15	Dark to light (D-L)	Cagemate	First trial dark, then light-light-dark. No food deprivation, average bodyweight fully recovered^c^ (range 0–3%)

^a^ The last three trials of the Food and Empty condition were used for analysis.

^b^ Three rats did not increase latency to open when the restrainer was empty. These three were tested with an empty restrainer for one additional day and did not complete Trapped cagemate Day 2 (session 13).

^c^ Same weight as at age 114 days.

Eight students assisted in the experiments, and each of the rat-couples was assigned one set of handlers for the entirety of the experiment; two student laboratory assistants and the primary author. To minimize noise in the data, a detailed experimental protocol was developed, and all students underwent a training program in animal handling and experimental testing under the auspices of the main author. The laboratory assistants were continuously supervised by the main author to ensure that protocols for laboratory conduct and experimental procedures were followed. The following measures were taken to reduce effects of single housing and food deprivation when proceeding to subsequent conditions: 1) The rats were allowed to play and socialize for 1 h each day in a separate cage and were housed in adjacent cages to maintain social bonds, and, 2) 60 h of co-habitation and food *ad lib* took place before proceeding to the next condition.

### Measures

**Fig 2:**
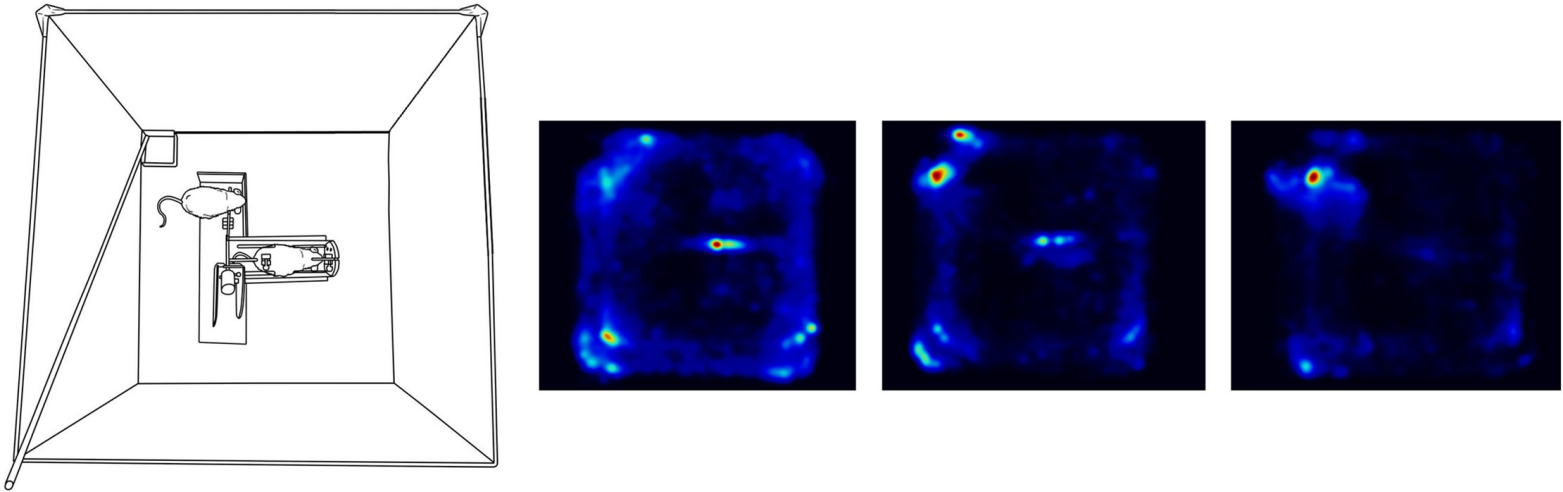
From left to right: Overview of the arena, day 1, day 4 and the last day (7) of shaping. Rats were given food reinforcement for spending time in the quadrant (top left corner) of the box containing the opening mechanism, door, and food box. Red/bright blue areas indicate areas where the rats spent the most time during the trials.

To assess the effects of shaping, video-recordings of behaviour during session one, four and seven of were analysed using the Ethovision XT software package (Noldus, Netherlands). Heat maps (Fig 2) show how behaviour gradually changed following the shaping procedure. The first day, the rats explored the arena and spent most time close to the walls, the corners, and around the restrainer. By day four of behavioural shaping, the movement had become more restricted to the area where food pellets were delivered. On the seventh day of shaping, the rats spent most of the time in the top left corner of the arena.

We measured latency to open the restrainer door, opening topography (technique), and occurrence of aggressive behaviour after opening. All measures were obtained by manually inspecting the video recordings with VLC media player (VLC Mediaplayer, VideoLAN) on a Microsoft Windows computer.

Latency was defined as the time from the rat was inserted into the arena, with no part of the experimenter visible on the video-screen, until the opening of the restrainer door. In all conditions, there was limited time available for restrainer door opening before the trial was terminated. The time limit (maximum latency) was 5 min during the Food and Empty conditions (sessions 8–11), and 10 min in the other conditions (sessions 12–15). In case no door opening occurred within the time limit, latency was set to maximum (i.e., 5 min) to enable quantitative comparisons across conditions. Max latency was higher during pro-social testing to give ample time for cage mate release to occur, as this was the main focus of the study. Although we used different max latencies across conditions, a latency score of 300 has a comparable meaning in all conditions; no opening took place during the first 5 minutes. Still, the different max latencies across conditions introduces a possible ambivalence in interpreting the results because the consequence of not opening was different across conditions (i.e. the animal was left in arena for 5 or 10 min).

The rats could use one of three response topographies (door-opening techniques) to open the restrainer door: Pressing the lever, tipping the door open with paws/head, or tipping over the counterweight. In the shaping procedure, only lever pressing was reinforced. However, in the following condition when the restrainer was baited with food, the rats started to tip the door open using their paws or head. Although this response topography was not reinforced during the shaping procedure, it served the same function as lever presses and consequently belong to the same operant class [52]. Due to the change in response topography prior to testing with a trapped cagemate, the opening topography used by the animals in the Food condition termed “Food-reinforced openings” was used for scoring correspondence with opening topography in the subsequent conditions with a trapped cagemate.

Aggressive behaviours were defined as instances of biting or pinning, during the interplay between the rats following the release of the trapped cagemate.

### Statistical analyses

The study employed a within-subjects design, in which all rats underwent the experimental procedure in the same order (Table 1) with a few exceptions. Three rats were exposed to one additional Empty session due to short latencies during the first Empty session. These short latencies could perhaps be explained by prolonged food deprivation effects even though the weight was regained at this point (Table 1). For these rats, this additional session was used in the analyses, and they also did not complete Trapped cage mate Day 2 where imputations were made (below). Analyses of these three rats separately showed latencies that did not contribute differently than the other rats in any condition. Therefore, all animals were included in the following statistical analyses.

Each session included four trials. The first trial was excluded from the statistical analyses for two reasons: 1) Prior to testing, the animals were moved from the sleeping quarters to the experimental room. During transport, they were exposed to sounds and smell in addition to shaking and movement that was suspected to affect test results, and 2) During some conditions (e.g. empty, the first with trapped cage mate), the first trial was the first time the animals came in contact with the new contingencies; the consequence of door opening would have had no previous opportunity to affect behaviour.

Average door opening latencies across the conditions were analysed with a non-parametric Friedman test, followed by multiple pairwise comparisons using Nemenyi's procedure / Two-tailed test. Wilcoxon signed-rank test / Two-tailed test was used to compare door opening latencies in the dark vs light conditions. An alpha level of 0.05 was used for all statistical tests, and all statistical analyses were performed with Xlstat (Addinsoft, 2019).

### Imputations and outliers

In order to run the Friedman test, 18 (7.1%) missing trial data points were replaced by imputations. For three rats in the Trapped cagemate Day 2 condition, missing data from nine incomplete trials were replaced. Additionally, mechanical failure led to loss of one data point in Trapped cagemate Day 2, five in condition L-D and three in condition D-L.

Visual inspection indicated one or two outliers in some of the conditions in the dataset. To investigate this, we ran double Grubbs test. The double Grubbs test revealed one outlier in condition Food, Trapped cagemate Day 1, Trapped cagemate Day 2, and DL, respectively. These outliers were removed and imputed. All trial and average imputations were made with the MD Imputation function in Statistica (Statsoft Inc., 2014) which uses the k-nearest neighbour algorithm.

## Results

### Number of trials to the first door opening, topography, and behaviour following door opening

In the present study, door opening was first shaped and then reinforced by food to ensure that the behaviour was in the animal’s repertoire before testing with a trapped cagemate. The first occurrence of door opening to release a cagemate was observed in 12 out of 14 (85%) subjects in trial one during day 1, and 13 out of 14 (≈93%) rats opened in at least one out of the three trials.

The rats employed the food-reinforced opening topography 77.9% of the time across all conditions with a trapped cagemate. Inter-observer agreement for opening topography was 95% across the conditions Food, Trapped cagemate Day 1 & 2, L-D and D-L.

Visual observations of the recorded interplay between the rats following the release of the trapped cagemate revealed no instances of biting, pinning or other aggressive behaviours in any sessions and will not be discussed further.

### Door opening latency across conditions

**Fig 3:**
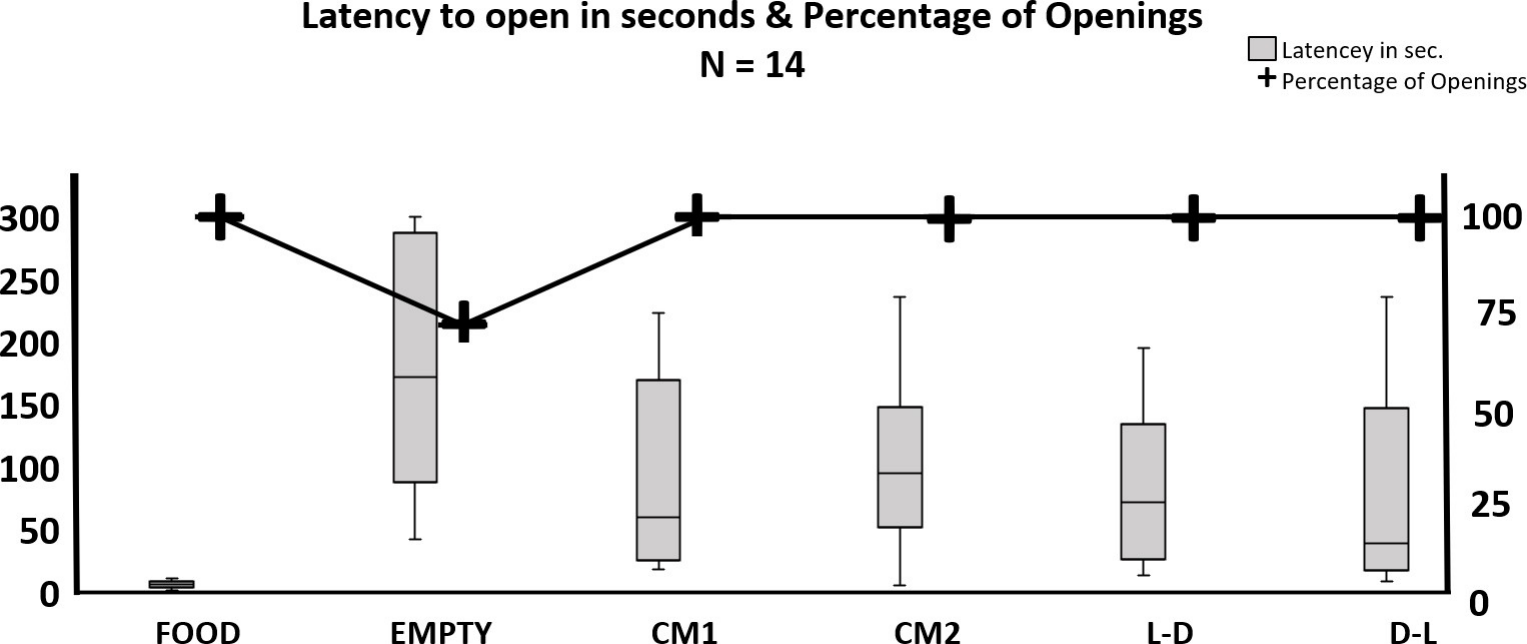
Door opening (boxplot) and percentage of openings (solid line) across the experimental conditions (left and right ordinate, respectively). Door opening latency based on mean of three trials was shorter when the restrainer contained a trapped cagemate than when it was empty but longer than when the restrainer contained food. The box whiskers extend to the most extreme data points lying within 1.5 interquartile range. The percentage of openings were calculated by number of subjects that opened the restrainer door at least once during the condition, divided by total number of subjects.

Door opening latency was shortest under the Food condition, longest during the Empty condition, and of intermediate duration in the remainder conditions (Fig 3).

The comparisons of conditions were done with was done with a non-parametric Friedman test. The Friedman test rendered a Chi square value of 42.122, which was significant (p>0.0001). Multiple pairwise comparisons using Nemenyi's procedure / Two-tailed test was run after the significant Friedman test to investigate which conditions were different from each other. The Nemenyi’s procedure showed that the Food condition was statistically significantly different from all other conditions except the DL illumination condition where the *p*-value was just shy of significance (*p* = 0.056); the Empty condition was statistically significantly different from all other conditions except the CM2 condition; and none of the four conditions with a trapped cagemate were statistically significantly different from each other (see Table 2).

**Table 2:** Nemenyi’s comparisons of condition.

	FOOD	EMPTY	CM1	CM2	LD	DL
**FOOD**	0	**-4.500**	**-2.357**	**-2.857**	**-2.429**	-2.000
	*p* = 1	**p < 0.0001**	**p = 0.0013**	**p = 0.001**	**p = 0.009**	p = 0.056
**EMPTY**	**4.500**	0	**2.143**	1.643	**2.071**	**2.500**
	**p < 0.0001**	p = 1	**p = 0.032**	p = 0.189	**p = 0.043**	**p = 0.006**
**CM1**	**2.357**	**-2.143**	0	0.500	0.071	0.357
	**p = 0.013**	**p = 0.032**	p = 1	p = 0.981	p = 1.000	p = 0.996
**CM2**	**2.857**	-1.643	0.500	0	0.429	0.857
	**p = 0.001**	p = 0.189	p = 0.981	p = 1	p = 0.991	p = 0.831
**LD**	**2.429**	**-2.071**	0.071	0.429	0	0.429
	**p = 0.009**	**p = 0.043**	p = 1.000	p = 0.991	p = 1	p = 0.991
**DL**	2.000	**-2.500**	0.357	0.857	0.429	0
** **	p = 0.056	**p = 0.006**	p = 0.996	p = 0.831	p = 0.991	p = 1

Critical difference: 2.0314

**Fig 4:**
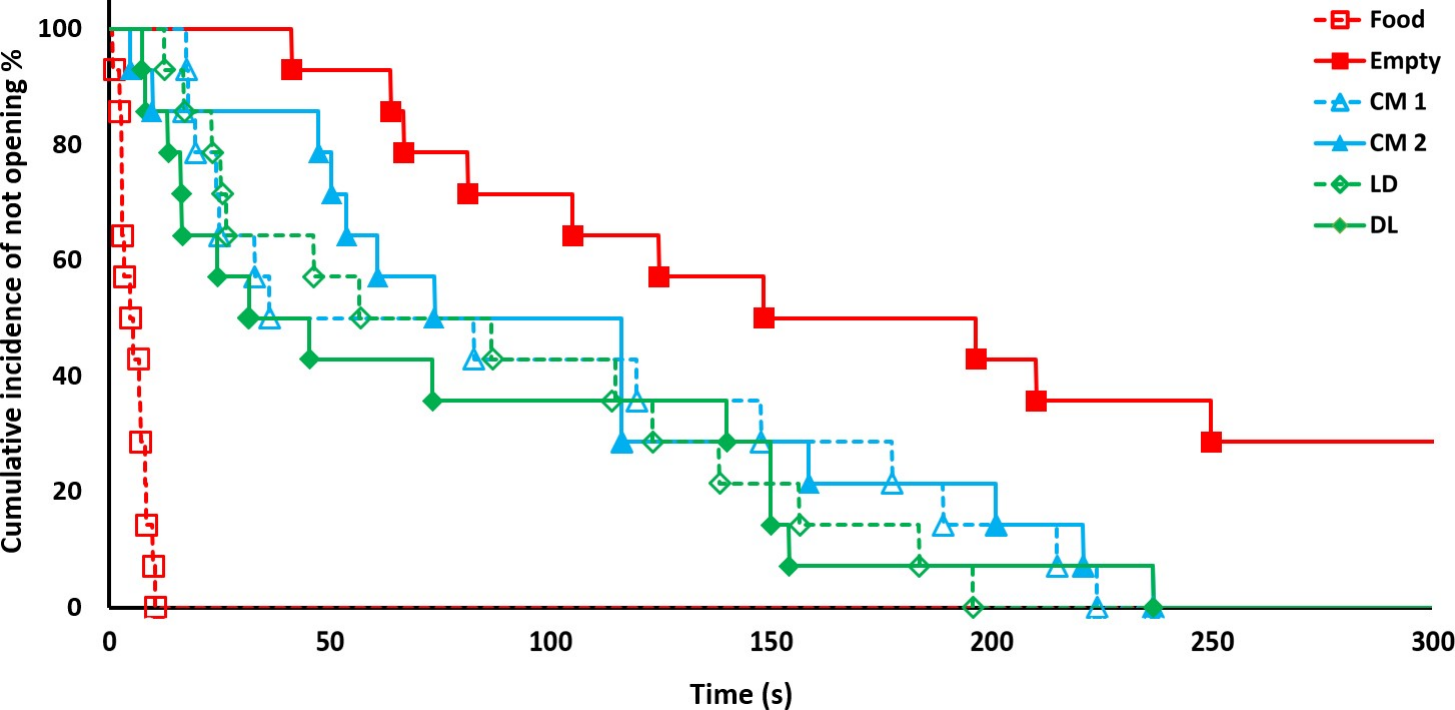
Cumulative incident plot showing percentage of not opening across time. All conditions with a trapped cagemate (CM1 through DL) show a decline with a steepness in-between the Food and Empty conditions. Each step down represents rat(s) opening the restrainer in the different conditions and are based on subject mean latency of the three trials in each condition.

Door-opening occurred in 100% of the trials except when the restrainer was empty where opening occurred in only 71.4% of the trials. (Fig 3, solid line). The cumulative incident plot (Fig 4) illustrates the different opening latencies with individual door openings represented as steps in the lines.

**Fig 5:**
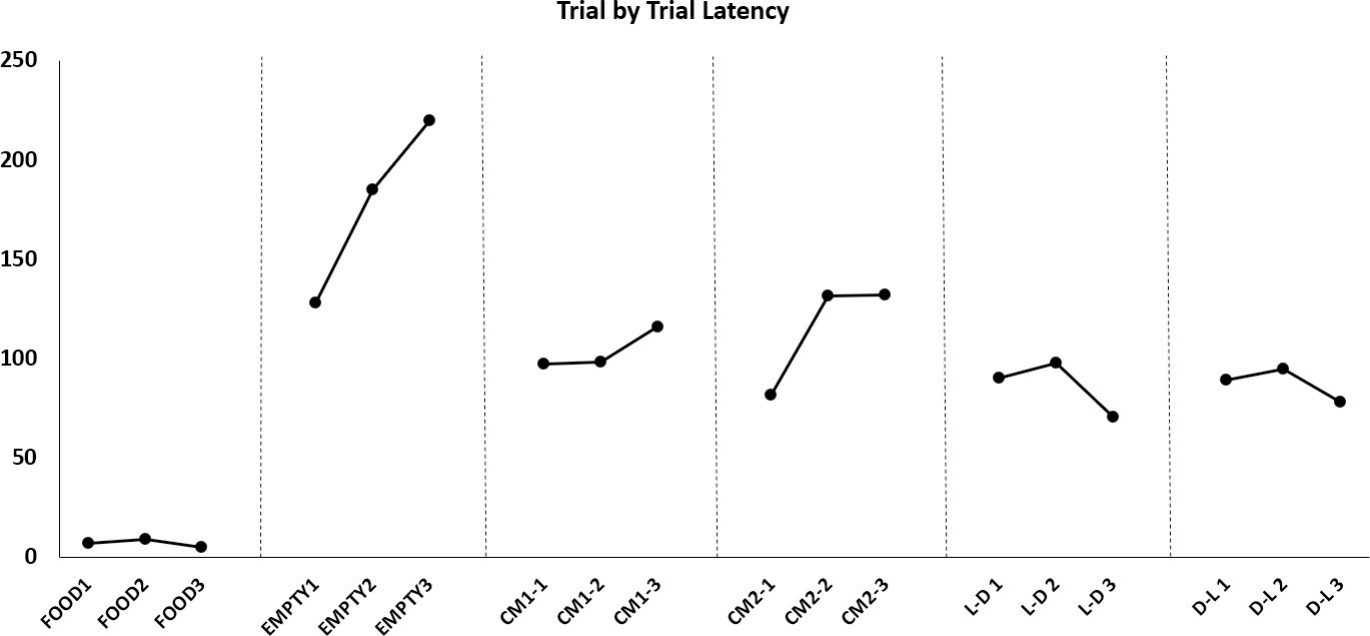
Average data illustrated in Figs 3 and 4 broken down into individual trials. The Food condition had the lowest and most stable latency, the Empty condition had a rising latency across trials, and the conditions where the restrainer contained a cagemate had a semi-stable pattern with an intermediate latency compared to Food and Empty.

In order to visualize possible differences on a trial-by-trial level, the average score for each trial within each condition was graphed. Food showed a stable low latency, Empty showed a rising high latency, and all condition with a trapped cagemate had semi-stable, intermediate latency scores (see Fig 5).

### Illumination: No significant effects

Light and dark sessions were compared in order to investigate effects of illumination. The average latencies were 89.9 s for dark, and 82.7 s for light. A non-parametric Wilcoxon signed-rank test / Two-tailed test did not reveal any significant differences between sessions in the light vs in the dark (p>0.05).

## Discussion

The present experiment studied door opening to release a trapped cagemate using a modified procedure developed by Bartal et al. (2011) which included demonstration of door opening and baseline recordings before testing with a trapped cagemate. Additionally, the effects of illumination on door opening were examined. The findings show that door opening topography to release a cagemate was largely the same as when the restrainer was baited with food. The first occurrence of restrainer door opening to release a trapped cagemate took place during the first few trials. Door opening latency was shortest when the restrainer contained food, intermediate when the restrainer contained a cagemate and longest when the restrainer was empty (see Figs 3–5). Door openings occurred in 100% of the trials when the restrainer contained food or a cagemate, and in 71.4% of the trials when the restrainer was empty (Fig 3, solid line). Finally, door opening latencies to release a cagemate was not different across the two levels of illumination when all trials were analysed.

### Door opening—Shaping, first occurrence, and topography

In the present study, door openings were shaped before door opening latencies when the restrainer was empty or contained food were examined. This procedure served three main functions. First, by explicitly training door-opening, the acquisition of this behaviour was not left to chance but was controlled by the experimenter. In previous studies, no shaping procedure was in effect [21,41]. Thus, the occurrence of the first door-opening in these experiments was not experimenter-controlled, but spontaneously emitted by the rat as it roamed around freely and explored the arena. Second, the inclusion of the two control conditions where the restrainer was either empty or contained food provided essential baseline comparisons for interpreting latencies during later testing with a trapped cagemate. Third, latency comparisons across these conditions reveal the degree and relative strength to which the restrainer content control door opening.

In the present study, food was used to train door opening in all rats before testing with a cagemate. Sato et al. (2015) reported that the stimulus used to shape door opening in rats (food or helping a soaked cage-mate) affected subsequent choice. They found a higher proportion of pro-social behaviour in the group trained to open the door for a soaked cage-mate compared to the group trained to open for food reinforcers. However, when the two groups were presented with the choice between opening one door leading to food and another door letting the soaked cage mate out of the pool area, they found that first-choice latency was not different in the two groups. The implication for the present study is that learning door opening in the context of a trapped cagemate may be different from learning door opening through a shaping procedure with food reinforcers, thus, type of reinforcer used during the shaping procedure may affect the data.

Across the seven days of shaping, the rats spent more time in the area containing the lever opening the door, around the door itself, and by the food delivery box (Fig 2). Subsequent shaping, all rats demonstrated proficiency to open the door to obtain food and did so on 100% of the trials with short opening latencies. However, during this condition, a shift in response topography was observed. Lever pressing, reinforced during the shaping procedure, was replaced by tipping the door open by the paws or head. This change in response topography may have occurred because some response topographies are more similar to natural behaviour than other responses [53]. If some responses are easier to learn and maintain than others, this may also affect response rates or latencies used to assess effects of variables and conditions and have implications for the experimenter’s choice of response to release the trapped rat in experimental studies (e.g. lever press, nose poke) and for comparisons of data (i.e. latencies) across studies. It also emphasizes the importance of including a training procedure to ensure that the response is in the rat’s repertoire prior to testing with a trapped cagemate. In the present study, the response topography to access food in the restrainer was the same as when opening the door to release the cagemate (77.9% correspondence). In the present study where door openings were shaped, the first occurrence of restrainer door opening to release a trapped cagemate took place during the first few trials. In Bartal’s study, where no shaping procedure was included, one week of testing was needed before door opening was observed (Bartal et al., 2011). This difference is likely due to the pre-training procedure used in the present study. Without pre-training, the first occurrence of door opening is not controlled by the experimenter but emitted by the animal as part of exploration or general locomotion. Thus, responses not frequently found in the behavioural repertoire may take many trials to spontaneously occur. In Bartal’s study [21], this introduced a long and uncontrolled learning history, both for the trapped as well as the free rat, that may have affected the data. This could have been avoided if a pre-training procedure had been included but had the advantage that the response was learned in a social context, which may be of importance (Sato et al. 2015).

### Opening latency and percentage openings across conditions

The comparisons across conditions of door opening latencies and percentage of trials containing door-openings showed that restrainer content affected both measures (Fig 3). The latency was shorter, and a higher percentage of trials contained door-openings when the restrainer contained a cage-mate as compared to when the restrainer was empty. This difference indicates that some aspect(s) of freeing the trapped rat acted as a reinforcer(s) for the free rat’s door-opening. Previous findings suggest that several stimuli in the experimental setting may reinforce door openings, e.g. social contact or water [41,54]. The reinforcing stimuli in the present experiment could be several of these, but the present study was not designed to isolate and identify these reinforcers.

It may be argued that the data can be explained in terms of an extinction process or as a “transferred situation even when the outcomes are different”. Extinction is by definition the discontinuation of the reinforcement of a response, e.g. door opening; in the present study defining all trials following the Food condition. Studies show that during extinction, rate of previously reinforced responses returns to operant level, i.e. the level observed before responses were reinforced. General findings show that the extinction curve is a gradual decline in responding but may also initially include an extinction burst (the organism “tries harder” for a period) before rate of responding declines. The pattern of decreasing and then increasing latencies found for the conditions following food reinforcement in the present study does not conform to known extinction curves and runs counter to extinction as an explanation of the findings. Further, the suggestion of “transferred situation” implies a similarity between the reinforcement condition (Food) and subsequent conditions (Empty, cagemate) and that some situations are more similar that others causing differences in results across conditions. Across the conditions in our study, most preceding stimuli are the same although we cannot rule out the possibility of smell- (food, trapped cagemate) and sound-differences (vocalizations by trapped cagemate) across conditions that may have influenced behaviour. However, this possibility disagrees with the suggestion of similarities between situations. The consequences for opening the restrainer door obviously differ across conditions. It is conceivable that when the restrainer contains a cagemate, this consequence is more similar to when the restrainer contains food than when it is empty, and that this similarity causes the rats to open the restrainer door with shorter latencies than when the restrainer is empty. This suggestion implies that door openings are maintained during all cagemate trials by similarity to the Food condition and not by some aspect of freeing the cagemate acting as a reinforcer. If this explanation were to be true, one would expect that the rats learn to discriminate between the two consequences (food, cagemate) and stop responding towards the last cagemate-trials (Figs 3 and 5). After all, the rats are obviously able to discriminate between when the restrainer contains food and when it is empty (Figs 3 and 5), and quickly learn to do so. The stable pattern of latencies observed across trials in our study (Fig 5) suggests that the data is not explained by similarity between situations.

The food-condition produced shorter latencies than when testing with a trapped cagemate. Reinforcer value is not a unitary concept, and there are several accepted measures of reinforcement value in the literature [55]. However, given that latency reflects reinforcer value, our results indicate that food has a higher reinforcer value than freeing a cage-mate. These results are at odds with Bartal et al.’s (2011) findings of similar latencies to open the restrainer door for chocolate chips and to free a cage-mate, and who concluded that reinforcer value for accessing chocolate chips and freeing a cage-mate was the same. Additionally, a key point in Sato et al. (2015) is the usage of a setup with a soaked cagemate, and in their study they found that opening to release a cagemate was the first choice more often than opening to access food. This seems to be at odds with our finding that the latency to open was much faster in the food condition than in three out of the four conditions with a trapped cagemate (CM1, DL and LD). However, crucially for food to serve as a reinforcer for behaviour is hunger, and unlike this study, the rats in Sato et al. (2015) did not undergo food deprivation. This seems to be the likely reason why the rats in our study showed a lower latency for food than for most of the conditions with a trapped cagemate and illustrates the importance of investigating factors that influence reinforcer strength.

Several procedural differences between the studies may explain the inconsistent findings. Of particular importance is that both the Bartal et al. (2011) and Sato et al. (2015) studies used chocolate chips as reinforcers for responding in undeprived rats, whereas we in the present study used standard rat food for responding in rats weighing no less than 85% of free-feeding weight. Reinforcer values are not fixed but depend on past and immediate learning history including satiation and deprivation (as discussed in [56]). It is likely or possible that the motivating operation of food deprivation used in the present study increased the reinforcer value of food up and above the value of freeing a cage-mate or the value of chocolate chips in undeprived rats. Thus, the conflicting findings in the three studies may illustrate limitations to external validity—i.e., that findings are limited to the specific experimental manipulations used, including reinforcer type (food, chocolate chips, water), reinforcer amount, and deprivation level.

Illumination was briefly investigated in this study but did not yield any significant effects. Neither light, nor dark, settings were associated with positive or negative effect on helping behaviour. However, this study used a setup where rats were habituated and tested in the brightest setting, and additionally the rats were tested during the light-phase of their day/night cycle. Due to these limitations, we cannot dismiss that a study with a larger group, and with a design that controls for light cycle and illumination during habituation, can discover effects of illumination on door opening for cagemate release. Preferably, this should be replicated with a between-group design in which one group is tested in the dark and the other in the light. This replication should also include behavioural and biological measures of stress.

## Summary and conclusion

The presents study investigated restrainer door opening in rats using the helping paradigm developed and described by Bartal et al. (2011), but with a few changes to the experimental procedure used in the original study. First, a shaping procedure was included to ensure that the door-opening response was in the rats’ behavioural repertoire prior to pro-social testing. Additionally, the rats were tested when the restrainer was empty or food-baited to establish essential baseline comparisons for interpreting percentage of openings and opening latency during pro-social testing.

In the present study, the first occurrence of pro-social door-openings was observed during the first few trials of testing with a trapped cagemate. In Bartal’s study (2011), it took approximately one week of testing before door opening was observed. This is likely due to differences in training and habituation procedures in our (shaping with food reward) and Bartal’s (2011) (always trapped rat, no direct shaping) studies.

Our data shows that the rats opened the restrainer door with shorter latencies to release a cagemate than when the restrainer was empty, but with longer latencies than when the restrainer contained food (Fig 3). This suggests that food is a more potent reinforcer to a food-deprived rat than releasing a cagemate is to a rat not deprived of food. The food deprivation procedure used in the present study is the probable explanation for the shortest opening latency found when the restrainer was baited with food, a finding that is somewhat at odds with Bartal’s findings (2011). A future study should investigate the effect of deprivation and choice between opening for a cagemate and opening for food. Choosing between food and releasing a cagemate when the rat is hungry would also give more indication regarding the reinforcing value of releasing a trapped cagemate.

Illumination was briefly investigated in this study but did not yield any significant effects. We suggest a proper between-group experiment with one group habituated and tested in the dark vs one group habituated and tested in the light to properly address this environmental variable. This should also include proper measures of stress, both biological and behavioural.

Trial-by-trial latencies indicate that stable state was not reached in all conditions, and this should be addressed in future research. Stable state behaviour has not been a point of focus in prior research either, with some papers only recording one response per day [21,41]. If a stable state was reached, this could possibly yield clearer results between Empty and other conditions in the present study, as the behaviour in Empty shows an increase in latency throughout trials (Fig 4).

There is a difference in total trial length across conditions, with 5 minutes for Food and Empty vs 10 minutes for the other conditions with a trapped cagemate. Even if the results show that most openings occurred within 5 minutes during conditions with a trapped cagemate (see for instance Fig 5), a theoretical possibility remains that this difference in trial length could affect the results. For that reason, we suggest retaining the same maximum time for future studies.

Oestrous cycle and possible associations between ovarian hormones and door opening latencies were not measured in our study. This is a limitation of the present and previous studies (e.g. [21,41,54]) of cage mate release, as hormone level is known to affect both social -, operant -, and open field behaviour in rats [57,58]. Future studies should include oestrous cycle and hormone level measurements to test how hormones affect cage mate release and for the generalizability of findings.

In conclusion, the main findings in the present study replicate and extend the findings in Bartal et al. (2011). Rats pre-trained to open the restrainer door for food will also open the door to release a cagemate, though with longer opening latencies than for food. Whether this opening behaviour is best conceptualized as empathically motivated, pro-social behaviour, or is motivated and controlled by social contact is debated and has yet to be resolved.
